# Examining the current health of Gulf War veterans with the veterans affairs frailty index

**DOI:** 10.3389/fnins.2023.1245811

**Published:** 2023-09-07

**Authors:** Linda L. Chao

**Affiliations:** ^1^Department of Radiology and Biomedical Imaging, University of California, San Francisco, San Francisco, CA, United States; ^2^Department of Psychiatry and Behavioral Sciences, University of California, San Francisco, San Francisco, CA, United States; ^3^San Francisco Veterans Affairs Health Care System, San Francisco, CA, United States

**Keywords:** Gulf War, Gulf War Illness, health symptoms, frailty, electronic frailty index, claims-based frailty index

## Abstract

**Introduction:**

Gulf War Illness (GWI) is a chronic, multisymptom (e.g., fatigue, muscle/joint pain, memory and concentration difficulties) condition estimated to affect 25–32% of Gulf War (GW) veterans. Longitudinal studies suggest that few veterans with GWI have recovered over time and that deployed GW veterans may be at increased risks for age-related conditions.

**Methods:**

We performed a retrospective cohort study to examine the current health status of 703 GW veterans who participated in research studies at the San Francisco VA Health Care System (SFVAHCS) between 2002 and 2018. We used the Veterans Affairs Frailty Index (VA-FI) as a proxy measure of current health and compared the VA-FIs of GW veterans to a group of randomly selected age- and sex-matched, non-GW veterans. We also examined GW veterans’ VA-FIs as a function of different GWI case definitions and in relationship to deployment-related experiences and exposures.

**Results:**

Compared to matched, non-GW veterans, GW veterans had lower VA-FIs (0.10 ± 0.10 vs. 0.12 ± 0.11, *p* < 0.01). However, the subset of GW veterans who met criteria for severe Chronic Multisymptom Illness (CMI) at the time of the SFVAHCS studies had the highest VA-FI (0.13 ± 0.10, *p* < 0.001). GW veterans who had Kansas GWI exclusionary conditions had higher VA-FI (0.12 ± 0.12, *p* < 0.05) than veterans who were Kansas GWI cases (0.08 ± 0.08) and controls (i.e., veterans with little or no symptoms, 0.04 ± 0.06) at the time of the SFVAHCS research studies. The VA-FI was positively correlated with several GW deployment-related exposures, including the frequency of wearing flea collars.

**Discussion:**

Although GW veterans, as a group, were less frail than non-GW veterans, the subset of GW veterans who met criteria for severe CDC CMI and/or who had Kansas GWI exclusionary conditions at the time of the SFVAHCS research studies were frailest at index date. This suggests that many ongoing studies of GWI that use the Kansas GWI criteria may not be capturing the group of GW veterans who are most at risk for adverse chronic health outcomes.

## 1. Introduction

More than 650,000 U.S. soldiers served in Operations Desert Shield and Desert Storm, the military campaign waged by a coalition force of 35 nations against Iraq in 1991 in response to the invasion and annexation of Kuwait. After the Gulf War (GW) ended, many soldiers returned home with a multitude of unexplained symptoms (e.g., fatigue, memory and concentration problems, muscle and joint pain, headaches, gastrointestinal and other health problems) (Freeman et al., 2020). Gulf War Illness (GWI) is the term commonly used to describe this condition, which is estimated to affect 25–32% of deployed GW veterans (White et al., 2016). Longitudinal research suggests few GW veterans have recovered or substantially improved from GWI in the decades since the Gulf War ended (Research Advisory Committee on Gulf War Veterans Illnesses (Rac-Gwvi), 2008, 2014; White et al., 2016).

Over the years, the cross-sectional studies that have examined GW veterans’ post-deployment health status have reported higher rates of chronic multisymptom illnesses such as fibromyalgia (Ismail et al., 2008; Kang et al., 2009), chronic fatigue syndrome (Unwin et al., 1999; Gray et al., 2002; Ismail et al., 2008; Kang et al., 2009; Li et al., 2011), irritable bowel syndrome (Gray et al., 2002; Kang et al., 2009), and chronic neurological disorders such as amyotrophic lateral sclerosis (Haley, 2003; Horner et al., 2008), brain cancer (Bullman et al., 2005; Barth et al., 2009; Young et al., 2010), seizures (Kang et al., 2000; McCauley et al., 2002; Kang et al., 2009), neuralgias and neuritis (Kang et al., 2000, 2009), and chronic migraine headaches (Unwin et al., 1999; Kang et al., 2000; Gray et al., 2002; Kelsall et al., 2004; Porter et al., 2018). There have also been reports of higher rates of arthritis (Unwin et al., 1999; Kang et al., 2000; Gray et al., 2002; Li et al., 2011; Porter et al., 2018), lung diseases (Unwin et al., 1999; Kang et al., 2000; Cowan et al., 2002; Gray et al., 2002; McCauley et al., 2002; Kang et al., 2009; Li et al., 2011), eye or vision problems (Kang et al., 2000) hypertension (Unwin et al., 1999; Kang et al., 2000; Gray et al., 2002; Ismail et al., 2008; Kang et al., 2009; Li et al., 2011), and heart disease (Kang et al., 2000; McCauley et al., 2002; Kang et al., 2009; Li et al., 2011; Porter et al., 2018) in GW veterans compared to other veteran control groups. Moreover, there is suggestive evidence that deployed GW veterans may be at significantly higher risks for age-related conditions (e.g., hypertension, hypercholesterolemia, myocardial infarct, diabetes, stroke, arthritis, and chronic bronchitis) compared to civilians (Zundel et al., 2019).

The first aim of this study was to examine the current health status of GW veterans who previously participated in research studies at the San Francisco VA Health Care System (SFVAHCS). We used the Veterans Affairs Frailty Index (VA-FI) (Orkaby et al., 2019; Cheng et al., 2021) as a proxy measure of aging and vulnerability to poor outcomes. Frailty is a clinical syndrome commonly described in older adults (Fried et al., 2001). Frailty can increase an individual’s vulnerability to adverse events such as mortality, morbidity, disability, and hospitalizations (Fried et al., 2001; Clegg et al., 2013), particularly after exposure to stressors (Morley et al., 2013). The VA-FI (Orkaby et al., 2019, Cheng et al., 2021) is an electronic index calculated from Veterans Health Administration (VHA) claims and electronic health records (EHR). The current study used the VA-FI as a proxy measure of the current health status of GW and non-GW veterans.

In the absence of diagnostic tests and validated case definitions for GWI, researchers have relied upon different criteria to study the chronic multisymptom condition. In 2013, the VA contracted with the Institute of Medicine (IOM), now the National Academy of Medicine (NAM), to develop a single case definition for GWI. However, the IOM/NAM panel was unable to do this due to limitations with the data that was available at the time (Institute of Medicine, 2014). Instead, the panel recommended the Kansas GWI case definition (Steele, 2000) be used for research and the Center for Disease Control and Prevention (CDC) Chronic Multisymptom Illness (CMI) case definition (Fukuda et al., 1998) be used for clinical purposes. However, both of these case definitions were developed in the late 1990s and early 2000s and were based on the types and pattern of symptoms reported by GW veterans in the decade post-deployment (Steele et al., 2021). Furthermore, because both case definitions ask veterans whether they experienced symptoms over the past 6 months, neither case definition captures or accounts for potential changes in GWI symptoms over time (Maule et al., 2018; Steele et al., 2021). Therefore, a second aim of this study was to examine the current health status of GW veterans as a function of the different GWI case definitions ascertained between 2002 and 2018.

Many GW veterans were exposed to a myriad of chemicals during deployment (White et al., 2016). Some of chemicals present in the GW milieu were neurotoxins that have since been associated with chronic health symptoms that encompass multiple body systems (Steele, 2000). Therefore, a final aim of this study was to examine the relationship between GW veterans’ deployment-related experiences and exposures and their current VA-FI.

To our knowledge, this is the first study to use the VA Frailty Index to examine GW veterans’ vulnerability to poor health outcomes. It has been suggested that deployed GW veterans may be aging more rapidly than their civilian counterparts (Zundel et al., 2019). For this reason, it is important to characterize and assess this group of veterans for the development of age-related syndromes. The major objectives of the study are to: (1) examine the VA-FI of past GW veteran research participants as a function of different GWI case definitions, (2) compare VA-FI in deployed GW veterans with a matched group of non-GW veterans, and (3) examine the relationship between VA-FI and GW veterans’ deployment related experiences and exposures. Because exposures to GW related chemicals and toxins may have altered the trajectory of aging in these veterans, it is important to have a better understanding of how GWI affects GW veterans’ current health and functional status.

## 2. Methods

All study procedures were approved by institutional review boards (IRB) at the University of California, San Francisco and the San Francisco VA Health Care System (SFVAHCS). Informed consent was obtained from all GW veterans who participated in the original research studies at the SFVAHCS. A waiver for consent was obtained for the retrospective cohort analysis because some of the study participants had died or were no longer receiving care through VHA when the analyses were performed.

We performed a retrospective cohort analysis of 703 GW veterans who participated in research studies related to GWI and GW-related exposures at SFVAHCS between 2002 to 2018. Electronic health records (EHR) and VA health care encounter diagnostic codes were identified for 602 of the 703 past GW veteran study participants in the VA’s corporate data warehouse (CDW).

The VHA is an integrated health care system that captures and stores claims and health care data in a centralized national database (Price et al., 2015). The VA-FI is an electronic frailty index calculated from VHA administrative claims and EHR (Orkaby et al., 2019; Cheng et al., 2021). We extracted *International Classification of Diseases* (*I*CD) diagnosis and procedure codes, Current Procedural Terminology (CPT), and Healthcare Common Procedure Coding System (HCPCS) codes for the GW veteran study participants from tables in CDW on an index date (04/05/2023). Smoking status was determined from the most recent clinical assessments of tobacco use prior to the index date.

Structured data in CDW and from the Death Ascertainment File indicated that 44 of the former SFVAHCS GW veteran participants were deceased at the index date. We calculated the VA-FI for the 588 GW veteran participants who were alive at an index date. The VA-FI was calculated as the proportion out of 31 equally weighted deficits incurred by the patient (Orkaby et al., 2019). These deficits account for impairments across multiple physiologic domains and were ascertained by the presence of specific sets diagnostic and procedure codes in patients’ records. We further classified the veterans as non-frail (VA-FI 0–0.1), pre-frail (VA-FI 0.1–0.2), mildly frail (VA-FI 0.2–0.3), and moderately frail (VA-FI 0.3–0.4) and severely frail (VA-FI >0.4), using previously published frailty index cutoffs (Armstrong et al., 2015; Blodgett et al., 2015; Pajewski et al., 2016).

To compare GW with non-GW veterans, we randomly selected 558 veterans from CDW who were matched in age and gender to the living GW veteran participants of SFVAHCS research studies. Because Congress never repealed the Authorization for Use of Military Force after the Gulf War ended in February 1991 (Department of Veteran Affairs, 2021), any military personnel who served on active duty from August 2, 1990 to present is considered a Gulf War veteran. Therefore, to identify non-GW veterans, we used a list of social security numbers obtained from the Defense Manpower Data Center (DMDC) of GW-deployed and non-deployed GW era veterans. Veterans were presumed to be non-GW veterans if their SSN was *not* on the DMDC list. We extracted ICD-10, CPT, and HCPCS codes from the CDW for non-GW veterans on the same index date.

### 2.1. Chronic Multisymptom Illness criteria

Chronic Multisymptom Illness (CMI) by the Center for Disease Control (CDC) criteria (Fukuda et al., 1998) was determined as the presence of persistent symptoms over 6 months in two out of three domains: fatigue, cognitive/mood, and musculoskeletal. Our measure of the CMI case definition included: one symptom in the fatigue domain (overly tired/lack of energy), four symptoms in the cognitive/mood domain (depressed mood, difficulty remembering, difficulty concentrating, trouble sleeping), and two symptoms in the musculoskeletal domain (joint pain and/or muscle pain). CMI was further categorized as “severe” if the veteran rated each defining symptom as severe. Otherwise CMI was categorized as “mild–moderate.” We used the veterans’ responses on the health questionnaire to ascertain CDC CMI case status.

### 2.2. Kansas Gulf War Illness (GWI) criteria

The Kansas GWI case definition requires veterans to have moderately severe or multiple chronic symptoms in at least three of six categories: fatigue/sleep problems, pain, neurological/cognitive/mood symptoms, respiratory complaints, gastrointestinal problems or skin symptoms (Steele, 2000). The Kansas GWI case criteria excludes veterans who have psychiatric conditions that may interfere with the accurate reporting of symptoms and/or medical conditions that might predict similar symptoms as GWI. We used the veterans’ responses on the Kansas Military History and Health questionnaire (Steele, 2000) to ascertain the Kansas GWI case status. Because this questionnaire was not part of all research protocols at the SFVAHCS, we were only able to ascertain Kansas GWI case status for a subset of the GW veteran study participants.

### 2.3. Khamisiyah exposure status

In March of 1991, U.S. troops detonated a munitions storage pit at Khamisiyah, Iraq that was later found to contain stockpiles of sarin and cyclosarin (Gulf Link, 2002). The plume that resulted from this demolition exposed potentially more than 100,000 U.S. troops to low levels of chemical nerve agents. Information about each veteran’s exposure to the Khamisiyah plume was obtained from the Deputy Assistant Secretary of Defense for Force Health Protection and Readiness, as previously described (Chao et al., 2010).

### 2.4. Other deployment-related exposures

We used the Kansas Gulf War and Health Questionnaire (Steele, 2000) to ascertain deployment-related exposures. The questionnaire asked veterans about a broad range of experiences and exposures specifically associated with Gulf War service. For each exposure that veterans endorsed, they had to indicate the duration of the exposure (i.e., 0 = no exposure; 1 = exposed for a week or less; 2 = exposed for 1 week to 2 month; 3 = exposed for a month or longer).

### 2.5. Statistical analyses

Mean values of continuous variables were compared using Wilcoxon rank sum test or the Kruskal-Wallis test. Proportional comparisons between veteran subgroups were assessed using chi- square tests. Spearman’s rank order correlation was used to examine the associations between VA-FI and deployment-related experiences and exposures. Previous reports indicate that many of the GW deployment-related exposures are highly intercorrelated (Fricker et al., 2000; Cherry et al., 2001; Steele et al., 2012). We also observed a high degree of correlation among the deployment related exposures (Spearman’s *ρ* = 0.13 to 0.58), which suggests the potential for confounding error when evaluating exposure associations individually. Therefore, in post-hoc analyses, we examined the relationship between deployment-related exposures and VA-FI with a backward-step linear regression model. Potentially confounding variables (i.e., index age and GWI case/control/exclude status) were also entered into the regression model with the deployment-related exposures that were significantly correlated with VA-FI. *p* values < 0.05 were considered significant. All analyses were performed using IBM SPSS Statistics (Version 29.0).

## 3. Results

### 3.1. Demographic and military characteristics

Table 1 summarizes the demographic and military characteristics of the GW veterans who participated in research studies at the SFVAHCS between 2002 to 2018. The analytic sample was comprised mostly of White males with 2–3 years of education post-high school who were approximately 63 years old at the index date. The sample was 13% women and 29% non-White.

**Table 1:** Demographics of the 704 Gulf War veteran participants.

	Analytic sample	Veterans not in CDW	Veterans with ERH in CDW		
			ALL	Alive @ index date	Deceased @ index date
*N*	703	101	602	558	44
Age (years) at time of SFVAHCS study participation	48.1 (10.2)	50.0 (11.1)	47.7 (10.0)	47.2 (8.9)	53.8 (10.7)
Age (years) at index date^a^ or death	62.5 (8.4)	64.4 (9.0)	62.2 (8.3)	62.2 (8.3)	66.1 (11.3)
Interval (years) between study participation and index date or death	14.6 (6.5)	14.3 (5.7)	14.7 (6.6)	14.9 (6.7)	12.3 (4.3)
Education (years)	14.9 (2.2)	15.4 (2.3)	14.8 (2.2)	14.8 (2.2)	15.5 (2.5)
Male: Female ratio	610:93	83:18	527:75	487:71	40:4
Race					
Caucasian^b^	416 (59.2%)	68 (67.3%)	348 (57.8%)	325 (58.2%)	23 (52.3%)
African American	85 (12.1%)	12 (11.9%)	73 (12.1%)	65 (11.6%)	8 (18.2%)
Other^c^	120 (17.1%)	8 (7.9%)	112 (18.6%)	108 (19.4%)	4 (9.1%)
Missing/no information	82 (11.7%)	13 (12.9%)	69 (11.5%)	60 (10.8%)	9 (20.5%)
SFVAHCS study participation status					
Included	551 (78.4%)	77 (76.2%)	474 (78.7%)	440 (78.9%)	34 (77.3%)
Excluded	61 (8.7%)	6 (5.9%)	55 (9.1%)	47 (8.4%)	8 (18.2%)
Withdrew	91 (12.9%)	18 (17.8%)	73 (12.1%)	71 (12.7%)	2 (4.5%)
GW Military Status					
Active duty	404 (58.0%)	46 (45.5%)	362 (60.1%)	343 (61.5%)	19 (43.2%)
Reserve	102 (14.5%)	23 (22.8%)	79 (13.1%)	69 (12.4%)	10 (22.7%)
National guard	37 (5.3%)	4 (4.0%)	33 (5.5%)	28 (5.0%)	5 (11.4%)
Missing/no information	157 (22.3%)	28 (27.7%)	128 (21.3%)	118 (21.1%)	10 (22.7%)
Predicted Khamisiyah exposure	156 (23.4%)	17 (16.8%)	148 (24.6%)	138 (24.7%)	10 (22.7%)
CDC CMI cases^d^	363 (51.5%)	44 (57.1%)	318 (66.9%)	297 (67.3%)	21 (61.8%)
CDC CMI status					
No symptoms – control	102 (14.5%)	19 (18.8%)	83 (13.8%)	77 (13.8%)	6 (13.6%)
Insufficient symptoms – control	88 (12.5%)	14 (13.9%)	74 (12.3%)	67 (12.0%)	7 (15.9%)
Mild-intermediate CMI	301 (42.8%)	38 (37.6%)	263 (43.7%)	248 (44.4%)	15 (34.1%)
Severe CMI	61 (8.7%)	6 (5.9%)	55 (9.1%)	55 (9.1%)	6 (13.6%)
No data^e^	151 (21.5%)	24 (23.8%)	127 (21.1%)	117 (21.0%)	10 (22.7%)

Data presented as mean (SD) or number (%). CDW, Corporate Data Warehouse; EHR, electronic health record. SFVAHCS, San Francisco Veteran Affairs Health Care System. CDC CMI, Centers for Disease Control and Prevention Chronic Multisymptom Illness.

a April 5, 2023.

b Does not includes White Hispanics.

c Includes Latino/Hispanic.

d Fatigue, muscle/joint pain, difficulty sleeping, remembering, concentrating, and feeling down depressed.

e Because participant was excluded or withdrew from study.

We located the EHR of 602 of the 703 former research study participants in CDW. Compared to veterans with EHR in CDW, veterans who were not in CDW were older at the index date (*Z* = 2.34, *p* = 0.02) and had more years of formal education (*Z* = 2.02, *p* = 0.04). There was marginally more veterans of “other” races among veterans in with EHR CDW than those not in CDW (*χ*^2^ = 7.26, *df* = 3, *p* = 0.06). More activity duty military personnel had EHR in CDW while more reservists did not have EHR in CDW (*χ*^2^ = 10.89, *df* = 3, *p* = 0.01).

**Figure 1:**
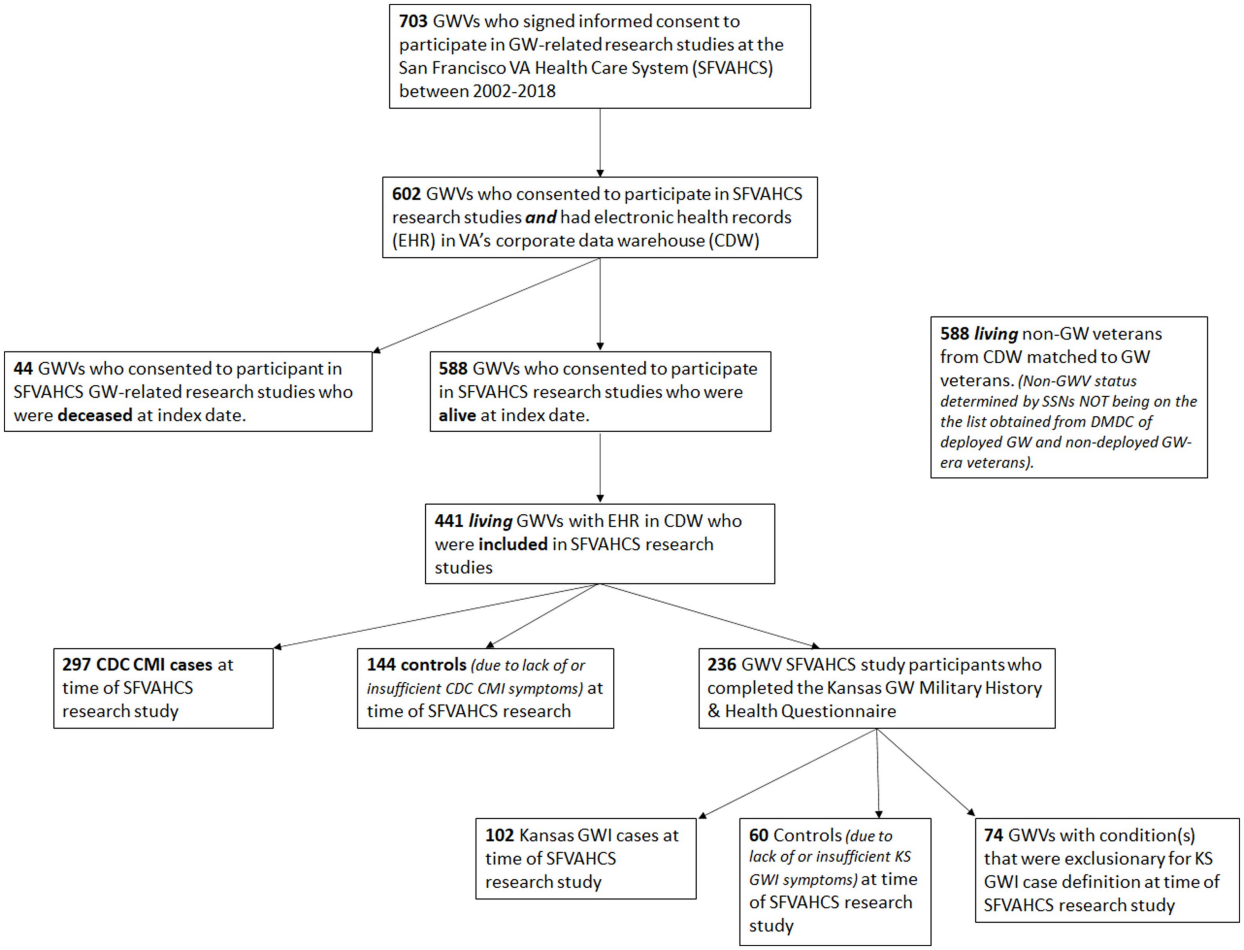
Schematic showing the total number of GW Veterans in the sample and how the various subgroups were derived. GWV, Gulf War Veterans; SFVAHCS, San Francisco VA Health Care System; ERH, Electronic Health Records; CDW, Corporate Data Warehouse; CDC CMI, Centers for Disease Control and Prevention Chronic Multisymptom Illness; GWI, Gulf War Illness; KS, Kansas.

Forty-four of the 602 past study participants with EHR in CDW were deceased at the index date. Compared to veterans who were alive at the index date, the veterans who had passed away were older at the time of the SFVAHCS research studies (*Z* = 3.85, *p* < 0.001). The average interval between the time of study participation and the index date was greater in living veterans compared to average interval between the time study participation and death among deceased GW veterans (*Z* = −3.71 *p* < 0.001). A larger proportion of veterans who were deceased at the index date had been in the Reserves during the GW (*χ*^2^ = 8.65, *df* = 3, *p* = 0.03). A larger proportion of veterans who were deceased at the index date had been excluded from the original SFVAHCS research studies (*χ*^2^ = 6.51, *df* = 2, *p* = 0.04). Figure 1 shows a schematic of how many veterans were in each sub-group for the different comparisons.

### 3.2. Effect of SFVAHCS study participation on VA-FI

Although all veterans initially signed informed consent forms to participate in research activities at the SFVAHCS, not all veterans were included in the studies. Some veterans were excluded because they did not meet all of the parent research studies’ inclusion criteria. Other veterans withdrew from the research studies after signing consents. There was a significant effect of study participation on VA-FI (H[2] = 13.77, *p* = 0.001): Veterans who were excluded from research had higher VA-FIs (0.15 ± 0.11) than veterans who were included (0.09 ± 0.01) and veterans who withdrew from the research studies (0.09 ± 0.09). There was a significant effect of study participation on VA-FI category (*χ*^2^ = 20.44, *df* = 8, *p* = 0.009). Fewer veterans who had been excluded from research were non-frail (40%) compared to veterans who had been included (65%) and veterans who withdrew (66%) from the research studies. At the same time, more veterans who had been excluded from research were mildly frail (21%) compared to veterans who had been included (8%) and veterans who withdrew (7%) from the research studies.

### 3.3. GW vs. non-GW veterans

**Figure 2:**
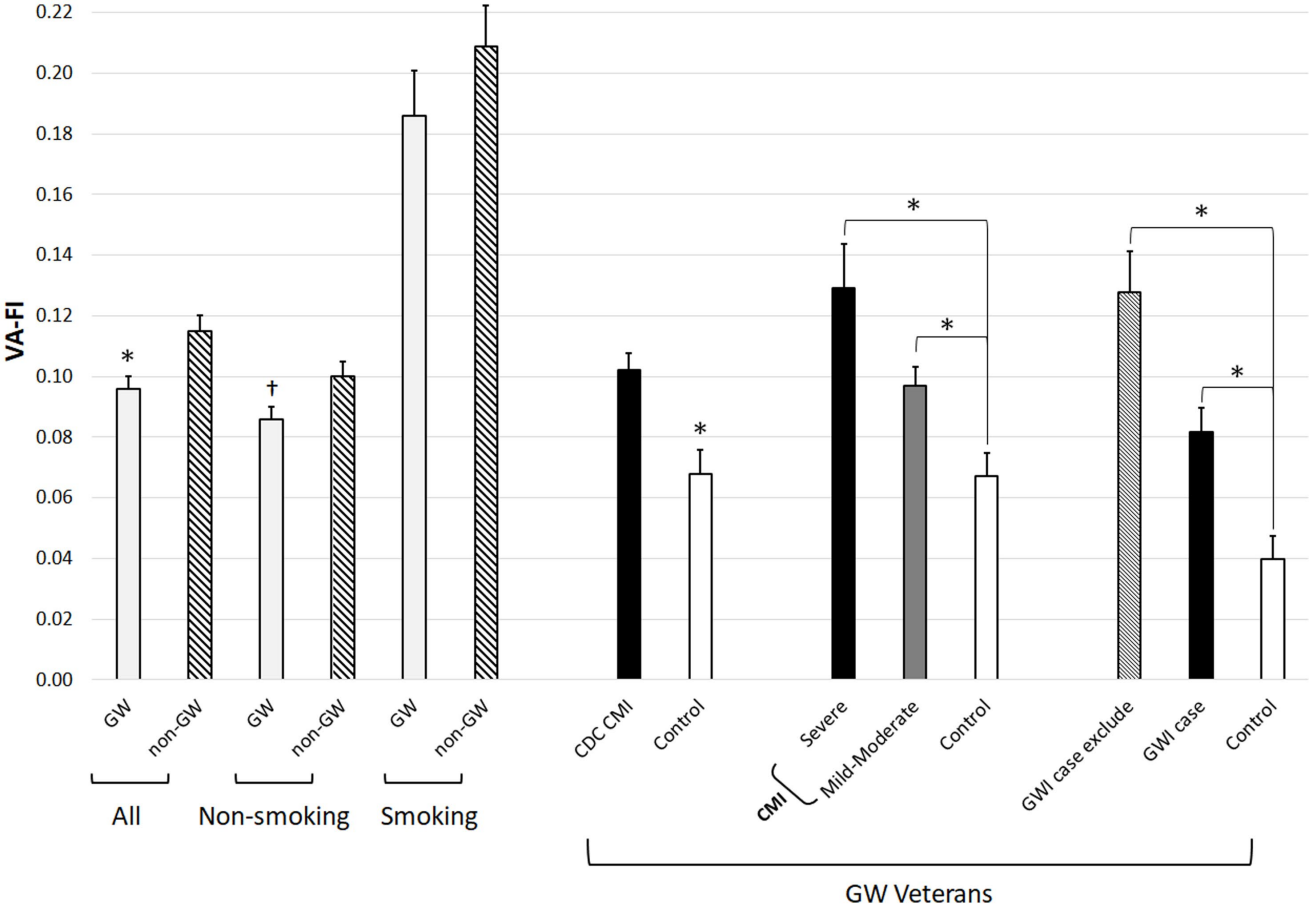
Bar graph showing VA-FI in GW and non-GW veterans as a function of current smoking status and in GW veterans as a function of CDC CMI and Kansas GWI case definition. Error bars represent standard error of the mean. ^*^p < 0.05, ^†^*p* = 0.06.

We randomly selected 588 veterans from CDW who were matched to the living GW veterans for age and sex (see Table 2). The mean VA-FI of the 558 GW veteran study participants who were alive at the index date was 0.10 ± 0.10 (see Table 3). Non-GW veterans had a higher VA-FI (0.12 ± 0.11) than GW veterans (*Z* = 2.84, *p* = 0.004). However, there were more current smokers among non-GW than GW veterans (*χ*^2^ = 6.48, *df* = 1, *p* = 0.01). When we re-ran the analysis and stratified by smoking status, the difference in VA-FI was only marginally significant in non-smokers (*Z* = 1.89, *p* = 0.06; GW: 0.09 ± 0.09; non-GW: 0.10 ± 0.10) and insignificant in smokers (*Z* = 1.04, *p* = 0.30; GW: 0.19 ± 0.11; non-GW: 0.21 ± 0.12; see Figure 2).

**Table 2:** Characteristics of GW and non-GW veterans.

	GW veterans	Non-GW veterans
*N*	588	588
Age (years) at index date, mean (SD)	62.1 (8.3)	62.2 (4.7)
Age range (years) at index date	50–87	54–72
Sex, % Male	487 (87.3%)	487 (87.3%)
Race, *n* (%)		
Caucasian	523 (58.2%)	340 (60.9%)
African American	65 (11.6%)	116 (20.8%)
Other*	108 (19.4%)	19 (3.4%)
Missing/no information	60 (10.8%)	83 (14.9%)
Current smokers, *n* (%)	55 (9.9%)	83 (14.9%)

*Includes Hispanic/Latino in GW veterans.

**Table 3:** VA-FI and individual health deficits in GW and Non-GW veterans.

	All		Current Non-Smokers Only	
	GWVs (*n* = 558)	Non-GWVs (*n* = 558)	GWVs (*n* = 503)	Non-GWVs (*n* = 475)
VA-FI	0.10 (0.10)	0.12 (0.11)*	0.09 (0.09)	0.10 (0.10)^†^
VA-FI categories				
Non-Frail (<0.1)	350 (62.7%)	311 (55.7%)	332 (66.0%)	291 (61.3%)
Pre-Frail (0.1–0.2)	133 (23.8%)	149 (26.7%)	118 (23.5%)	121 (25.5%)
Mildly Frail (0.2–0.3)	51 (9.1%)	59 (10.6%)	37 (7.4%)	40 (8.4%)
Moderately Frail (0.3–0.4)	18 (3.2%)	25 (4.5%)	12 (2.4%)	18 (3.8%)
Severely Frail (> 0.4)	6 (1.1%)	14 (2.5%)	4 (0.8%)	5 (1.1%)
VA-FI deficits				
Atrial fibrillation	29 (5.2%)	21 (3.8%)	25 (5.0%)	17 (3.6%)
Anemia	48 (8.6%)	61 (10.9%)	36 (7.2%)	38 (8.0%)
Anxiety	97 (17.4%)	130 (23.3%)*	78 (15.5%)	94 (19.8%)^†^
Arthritis	135 (24.2%)	162 (29.0%)^†^	108 (21.5%)	126 (26.5%)^†^
Coronary artery disease	32 (5.7%)	60 (10.8%)*	25 (5.0%)	36 (7.6%)^†^
Cancer	51 (9.1%)	49 (8.8%)	40 (8.0%)	42 (8.8%)
Chronic pain	98 (17.6%)	106 (19.0%)	78 (15.5%)	75 (15.8%)
Cerebrovascular disease	15 (2.7%)	33 (5.9%)*	14 (2.8%)	18 (3.8%)
Dementia	37 (6.6%)	27 (4.8%)	27 (5.4%)	18 (3.8%)
Depression	166 (29.7%)	171 (30.6%)	127 (25.2%)	123 (25.9%)
Diabetes	105 (18.8%)	141 (25.3%)*	86 (17.1%)	109 (22.9%)*
Durable medical equipment	66 (11.8%)	80 (14.3%)	56 (11.1%)	57 (12.0%)
Falls	14 (2.5%)	19 (3.4%)	8 (1.6%)	14 (2.9%)
Fatigue	49 (8.8%)	43 (7.7%)	38 (7.6%)	26 (5.5%)
Failure to thrive	1 (0.2%)	5 (0.9%)	0 (0%)	1 (0.2%)
Gait abnormality	34 (6.1%)	49 (8.8%)^†^	23 (4.6%)	33 (6.9%)
Hearing impairment/loss	103 (18.5%)	82 (14.7%)^†^	92 (18.3%)	70 (14.7%)
Heart failure	14 (2.5%)	22 (3.9%)	11 (2.2%)	13 (2.7%)
Hypertension	204 (36.6%)	268 (48.0%)*	174 (34.6%)	207 (43.6%)*
Incontinence	13 (2.3%)	18 (3.2%)	10 (2.0%)	13 (2.7%)
Kidney disease	31 (5.6%)	40 (7.2%)	25 (5.0%)	34 (7.2%)
Liver disease or cirrhosis	37 (6.6%)	32 (5.7%)	29 (5.8%)	24 (5.1%)
Lung disease (COPD, asthma)	83 (14.9%)	94 (16.8%)	67 (13.3%)	60 (12.6%)
Muscular issue	15 (2.7%)	25 (4.5%)	7 (1.4%)	13 (2.7%)
Osteoporosis	9 (1.6%)	13 (2.3%)	8 (1.6%)	10 (2.1%)
Parkinson’s disease	9 (1.6%)	10 (1.8%)	9 (1.8%)	9 (1.9%)
Peripheral neuropathy	37 (6.6%)	52 (9.3%)^†^	30 (6.0%)	39 (8.2%)
Peripheral vascular disease	27 (4.8%)	44 (7.9%)*	23 (4.6%)	30 (6.3%)
Thyroid disease	42 (7.5%)	43 (7.7%)	39 (7.8%)	35 (7.4%)
Vision comorbidity	47 (8.4%)	63 (11.3%)	40 (8.0%)	52 (10.9%)
Weight loss	13 (2.3%)	27 (4.8%)*	11 (2.2%)	15 (3.2%)

*Significantly (*p* < 0.05) different between GWV and non-GWV. ^†^Marginally (0.05 < *p* < 0.10) different between GWV and non-GWV.

There was a marginal effect of group on VA-FI category (*χ^2^* = 8.13, *df* = 4, *p* = 0.09). More GW than non-GW veterans had VA-FIs in the non-frail category (63% vs. 56%) and there was a trend for more non-GW than GW veterans to have VA-FIs in the severely frail category (3% vs. 1%). When we stratified the analysis by smoking status, there were no significant differences in VA-FI categories between GW and non-GW veterans among smokers and non-smokers.

The prevalence of individual VA-FI deficits in GW and non-GW veterans is presented in Table 3. Compared to non-GW veterans, GW veterans had lower rates of anxiety (*χ*^2^ = 6.02, *p* = 0.01), coronary artery disease (*χ*^2^ = 9.29, *p* = 0.002), cerebrovascular disease (*χ*^2^ = 7.05, *p* = 0.008), diabetes (*χ*^2^ = 6.76, *p* = 0.009), hypertension (*χ*^2^ = 15.04, *p* < 0.001), peripheral vascular disease (*χ*^2^ = 4.35, *p* = 0.04), and weight loss (*χ*^2^ = 5.08, *p* = 0.02). When we limited the analyses to current non-smokers, only differences in rates of diabetes (*χ*^2^ = 5.24, *p* = 0.02) and hypertension (*χ*^2^ = 8.30, *p* = 0.004) remained significantly different between GW and non-GW veterans.

### 3.4. Effects of CDC CMI case status

Table 4 summarizes the demographic characteristics of CDC CMI cases and controls. There was no age difference between CDC CMI cases and controls (i.e., veterans with few or no CMI symptoms) at the time of the SFVAHCS research studies; however, CDC CMI cases older at index date (*Z* = −2.49, *p* = 0.01) despite there being a shorter time interval between study participation and index date among CMI cases than controls (*Z* = −2.17, *p* = 0.03). Controls had more years of formal education compared to CMI cases (*Z* = −3.94, *p* < 0.001).

**Table 4:** Demographics of GW veterans as a function of CDC CMI and Kansas GWI case status.

	CDC CMI status		Kansas GWI status		
	Control	CMI case	Control	GWI case	GWI exclude
*N*	144	297	60	102	74
Age (years) at time of SFVAHCS study	48.3 (10.0)	47.5 (8.6)	49.9 (8.7)	48.4 (7.7)	50.0 (10.2)
Age (years) at index date^a^	64.2 (9.0)	61.9 (8.1)	62.4 (7.3)	59.7 (7.4)	64.7 (9.8)
Interval between SFVAHCS study and index date (years)	15.8 (4.2)	14.4 (5.1)	6.7 (1.0)	6.7 (1.0)	6.5 (1.0)
Education (years)	15.5 (2.3)	14.5 (2.1)	15.5 (2.4)	14.6 (2.3)	14.8 (2.2)
Male: Female ratio	129:5	252:45	50:10	85:17	65:9
Race					
Caucasian^b^	95 (66.0%)	196 (66.0%)	48 (80%)	75 (73.5%)	41 (55.4%)
African American	22 (15.3%)	33 (11.1%)	3 (5%)	9 (8.8%)	10 (13.5%)
Other^c^	26 (18.1%)	68 (22.9%)	9 (15%)	18 (17.6%)	23 (31.1%)
SFVAHCS study participation status					
Included	143 (99.3%)	297 (100%)	60 (100%)	102 (100%)	74 (100%)
Excluded	1 (0.7%)				
GW military status					
Active duty	113 (78.5%)	227 (76.4%)	47 (78.3%)	82 (80.4%)	54 (73%)
Reserve	20 (13.9%)	49 (16.5%)	8 (13.3%)	14 (13.7%)	17 (23%)
National guard	9 (6.3%)	19 (6.4%)	5 (8.3%)	6 (5.9%)	3 (4.1%)
Missing/no information^e^	2 (1.4%)	2 (0.7%)			
Predicted Khamisiyah exposure	39 (27.1%)	78 (26.3%)	20 (33.3%)	40 (39.2%)	25 (33.8%)
CDC CMI cases^d^	–	297 (100%)	20 (33.3%)	83 (81.4%)	56 (75.7%)
Severe CMI case	–	49 (16.5%)	0 (0%)	15 (14.7%)	8 (10.8%)

Data presented as mean (SD) or number (%).

a April 5, 2023.

b Does not includes White Hispanics.

c Includes Latino/Hispanic.

d Fatigue, muscle/joint pain, difficulty sleeping, remembering, concentrating, and feeling down depressed.

e Because participant was excluded or withdrew from study.

There was a significant effect of CMI case status on VA-FI (*Z* = 4.17, *p* < 0.001): Veterans who met CMI criteria at the time of the SFVAHCS studies had higher VA-FI (0.10 ± 0.01) than controls (0.07 ± 0.09, Table 5). See Figure 2, there was also a significant effect of CMI case status on VA-FI category (*χ*^2^ = 13.31, *df* = 4, *p* = 0.01). Compared to controls, fewer CMI cases had VA-FIs in the non-frail category (59% vs. 76%) while more CMI cases had VA-FIs in the pre-frail (28% vs. 15%).

**Table 5:** VA-FI and individual health deficits as a function of CDC CMI and Kansas GWI status.

	CDC CMI status		Kansas GWI status		
	Control (*n* = 144)	CMI case (*n* = 297)	Control (*n* = 60)	GWI case (*n* = 102)	GWI exclude (*n* = 74)
VA-FI	0.07 (0.09)	0.10 (0.10)*	0.04 (0.06)	0.08 (0.08)*	0.12 (0.12)*
VA-FI categories					
Non-Frail	110 (76.4%)	175 (56.9%)	53 (88.3%)	72 (70.6%)	34 (45.9%)
Pre-Frail	21 (14.6%)	83 (27.9%)	5 (8.3%)	22 (21.6%)	25 (33.8%)
Mildly Frail	9 (6.3%)	27 (9.1%)	2 (3.3%)	7 (6.9%)	9 (12.2%)
Moderately Frail	3 (2.1%)	9 (3.0%)	0 (0%)	1 (1%)	5 (6.8%)
Severely Frail	1 (0.7%)	3 (1.0%)	0 (0%)	0 (0%)	1 (1.4%)
VA-FI deficits					
Atrial fibrillation	6 (4.2%)	18 (6.1%)	4 (6.7%)	1 (1%)	6 (8.1%)
Anemia	13 (9.0%)	23 (7.7%)	1 (1.7%)	5 (4.9%)	9 (12.2%)*
Anxiety	14 (9.7%)	54 (18.2%)*	4 (6.7%)	23 (22.5%)*	10 (13.5%)
Arthritis	30 (20.8%)	73 (24.6%)	7 (11.7%)	24 (23.5%)~	24 (32.4%)*
Coronary artery disease	7 (4.9%)	21 (7.1%)	2 (3.3%)	5 (4.9%)	10 (13.5%)*
Cancer	13 (9.0%)	26 (8.8%)	4 (6.7%)	9 (8.8%)	9 (12.2%)
Chronic pain	7 (4.9%)	66 (22.2%)*	4 (6.7%)	24 (23.5%)*	13 (17.6%)~
Cerebrovascular disease	2 (1.4%)	8 (2.7%)	1 (1.7%)	1 (1.0%)	3 (4.1%)
Dementia	1 (0.7%)	29 (9.8%)*	2 (3.3%)	8 (7.8%)	5 (6.8%)
Depression	21 (14.6%)	95 (32.0%)*	5 (8.3%)	32 (31.4%)*	26 (35.1%)*^†^
Diabetes	20 (13.9%)	60 (20.2%)	0 (0%)	11 (10.8%)*	29 (39.2%)*
Durable medical equipment	13 (9.0%)	35 (11.8%)	2 (3.3%)	11 (10.8%)	12 (16.2%)~
Falls	5 (3.5%)	6 (2.0%)	0 (0%)	0 (0%)	2 (2.7%)
Fatigue	6 (4.2%)	25 (8.4%)	1 (1.7%)	8 (7.8%)	7 (9.5%)
Failure to thrive	0 (0%)	0 (0%)	0 (0%)	0 (0%)	0 (0%)
Gait abnormality	7 (4.9%)	17 (5.7%)	1 (1.7%)	1 (1.0%)	7 (9.5%)^†^
Hearing impairment/loss	25 (17.4%)	54 (18.2%)	11 (18.3%)	22 (21.6%)	14 (18.9%)
Heart failure	4 (2.8%)	5 (1.7%)	0 (0%)	0 (0%)	2 (2.7%)
Hypertension	44 (30.6%)	117 (39.4%)~	11 (18.3%)	29 (28.4%)	39 (52.7%)*^†^
Incontinence	2 (1.4%)	5 (1.7%)	0 (0%)	2 (2%)	0 (0%)
Kidney disease	7 (4.9%)	18 (6.1%)	0 (0%)	4 (3.9%)	7 (9.5%)*
Liver disease or cirrhosis	5 (3.5%)	23 (7.7%)~	0 (0%)	5 (4.9%)~	10 (13.5%)*
Lung disease (COPD, asthma)	14 (9.7%)	53 (17.8%)*	4 (6.7%)	15 (14.7%)	8 (10.8%)
Muscular issue	4 (2.8%)	4 (1.3%)	0 (0%)	1 (1%)	2 (2.7%)
Osteoporosis	1 (0.7%)	6 (2.0%)	0 (0%)	1 (1%)	4 (5.4%)~
Parkinson’s disease	1 (0.7%)	4 (1.3%)	1 (1.7%)	0 (0%)	0 (0%)
Peripheral neuropathy	4 (2.8%)	25 (8.4%)*	0 (0%)	3 (2.9%)	9 (12.2%)*^†^
Peripheral vascular disease	5 (3.5%)	17 (5.7%)	1 (1.7%)	1 (1%)	9 (12.2%)*^†^
Thyroid disease	5 (3.5%)	25 (8.4%)~	2 (3.3%)	9 (8.8%)	5 (6.8%)
Vision comorbidity	13 (9.0%)	21 (7.1%)	4 (6.7%)	3 (2.9%)	10 (13.5%)
Weight loss	3 (2.1%)	7 (2.4%)	2 (3.3%)	0 (0%)	2 (2.7%)

^*^Different from controls, *p* < 0.05. ^†^Different from GWI cases, *p* < 0.05. ~marginally (0.05 < *p* < 0.10) different from controls.

CMI can be categorized as “severe” if the veteran rates each defining symptom as severe or “mild moderate” for milder complaints. Of the 287 CMI cases, 49 met criteria for severe CMI cases while the remaining 248 were mild–moderate CMI cases. There was a significant effect of CMI category on VA-FI (H[3] = 28.89, *p* < 0.001): Controls had the lowest VA-FI (0.07 ± 0.9), mild–moderate CMI cases had intermediate VA-FI (0.10 ± 0.10), while severe CMI cases had the highest VA-FI (0.13 ± 0.10). See Figure 2, there was a significant effect of CMI severity category on VA-FI category (*χ*^2^ = 26.67, *df* = 12, *p* = 0.009). Fewer severe CMI cases had VA-FIs in the non-frail category (43%) compared to controls (76%) and mild–moderate CMI cases (62%). More severe CMI cases had VA-FIs in the pre-frail category (39%) compared to controls (15%) and mild–moderate CMI cases (26%).

Table 5 summarizes the VA-FI individual deficits in CMI cases and controls. There were significant differences in rates of anxiety (*χ*^2^ = 5.32, *df* = 1, *p* = 0.02), chronic pain (*χ*^2^ = 21.16, *df* = 1, *p* < 0.001), dementia (*χ*^2^ = 12.58, *df* = 1, *p* < 0.001), depression (*χ*^2^ = 15.15, *df* = 1, *p* < 0.001), lung disease (*χ*^2^ = 4.97, *df* = 1, *p* = 0.03), and peripheral neuropathy (*χ*^2^ = 5.02, *df* = 1, *p* = 0.03). There were trends of higher rates of hypertension (*χ*^2^ = 3.27, *df* = 1, *p* = 0.07), liver disease (*χ*^2^ = 2.98, *df* = 1, *p* = 0.08), and thyroid disease (*χ*^2^ = 3.74, *df* = 1, *p* = 0.05) between CMI cases and controls (see Table 4).

### 3.5. Effects of Kansas GWI case status

Of the GW veterans with ERH in CDW, we were able to ascertain Kansas GWI case status for 240 veterans: 74 veterans had medical and/or psychiatric conditions that excluded them from being considered as Kansas GWI cases (GWI excludes), 106 veterans met Kansas GWI criteria (4 were deceased at the index date), while 60 were controls.

Table 4 summarizes the demographic characteristics of GW veterans who met the Kansas GWI case condition and those who did not because they had few or insufficient GWI conditions (controls) or because they had conditions that were exclusionary for the Kansas GWI case definition. Although there was no significant age difference at the time of study participation, there was a group effect on age at the index date (H[3] = 13.43, *p* = 0.002) because GWI excludes were older than GWI cases. There was also a group effect on the interval between study participation and the index date (H[3] = 65.35, *p* < 0.001). There was a group effect on race on (*χ*^2^ = 11.04, *df* = 4, *p* = 0.03) because more controls were White while more GWI excludes were Black and other races. Fewer controls met CDC CMI criteria (*χ^2^* = 44.98, *df* = 2, *p* < 0.001) while no controls met severe CMI criteria (*χ*^2^ = 9.52, *df* = 2, *p* = 0.009).

There was a significant effect of Kansas GWI case status on VA-FI (H[2] = 27.37, *p* < 0.001): Controls had the lowest VA-FI (0.04 ± 0.6), Kansas GWI cases had intermediate VA-FI (0.08 ± 0.08), while Kansas GWI excludes had the highest VA-FI (0.13 ± 0.12; see Table 5 and Figure 2). Table 5 also summarizes the VA-FI individual deficits in GW veterans who were Kansas GWI cases, controls and had Kansas GWI exclusionary conditions. There was a significant effect of GWI case status on VA-FI (H[3] = 32.22, *p* < 0.001; controls, GWI cases, and GWI excludes were all significantly different from each other). There was a significant effect of GWI case status on VA-FI category (*χ*^2^ = 32.26, *df* = 8, *p* < 0.001): More controls were non-frail, more GWI cases and GWI excludes were pre-frail, and more GWI excludes were moderately frail.

Examination of VA-FI individual deficits revealed significant differences in anemia (*χ*^2^ = 6.77, *df* = 2, *p* = 0.03; GWI excludes > controls), anxiety (*χ*^2^ = 7.59, *df* = 2, *p* = 0.02; GWI > controls), arthritis (*χ*^2^ = 8.00, *df* = 2, *p* = 0.02; GWI excludes > controls, GWI marginally > controls), coronary artery disease (*χ^2^* = 6.56, *df* = 2, *p* = 0.04; GWI excludes > controls and marginally > GWI), chronic pain (*χ*^2^ = 7.49, *df* = 2, *p* = 0.03; GWI > controls, GWI excludes marginally > controls), depression (*χ*^2^ = 14.17, *df* = 2, *p* < 0.001; controls < GWI and GWI excludes), diabetes (*χ*^2^ = 41.00, *df* = 2, *p* < 0.001; all groups different from each other), gait abnormality (*χ*^2^ = 9.42, *df* = 2, *p* = 0.009; GWI excludes > GWI), hypertension (*χ*^2^ = 19.63, *df* = 2, *p* < 0.001; GWI excludes > GWI and controls), kidney disease (*χ*^2^ = 6.89, *df* = 2, *p* = 0.03; GWI excludes > control), liver disease (*χ*^2^ = 10.80, *df* = 2, *p* = 0.005; GWI excludes > control, marginally > GWI, GWI marginally > controls), peripheral neuropathy (*χ*^2^ = 11.87, *df* = 2, *p* = 0.003; GWI excludes > GWI and controls), and perivascular disease (*χ*^2^ = 13.69, *df* = 2, *p* = 0.001; GWI excludes > GWI and controls). There were marginal differences in rates of atrial fibrillation (*χ*^2^ = 5.63, *df* = 2, *p* = 0.06), use of durable medical equipment (*χ*^2^ = 5.81, *df* = 2, *p* = 0.06), and osteoporosis (*χ*^2^ = 5.79, *df* = 2, *p* = 0.06).

### 3.6. Relationship between deployment-related exposures and VA-FI

Table 6 lists the correlation coefficient between deployment-related exposures and experiences and VA-FI. Hearing Chemical alarms sound (Spearman’s *ρ* = 0.14, *p* = 0.03), using powdered pesticides on the skin, (Spearman’s *ρ* = 0.14, *p* = 0.03), wearing flea collars (Spearman’s *ρ* = 0.19, *p* = 0.005), and seeing living areas sprayed or fogged with pesticides (Spearman’s *ρ* = 0.14, *p* = 0.03) were significantly associated with VA-FI. Coming into contact with fresh chemical agent resistant coating (CARC) paint was marginally associated with VA-FI (Spearman’s *ρ* = 0.13, *p* = 0.05).

**Table 6:** Correlation Coefficients between VA-FI and deployment experiences/exposures.

GW experiences/exposures	Spearman’s *ρ*
Regular smoker during deployment	0.12
Saw smoke from oil well fires	−0.02
Heard chemical alarms sound	0.14*
Within 1 mile of exploding SCUD	0.08
Contact with prisoners of war	0.07
Contact dead animals	0.05
Contact with destroyed enemy vehicles	0.04
Contact with American vehicles hit by friendly fire	0.04
Used cream/spray pesticides on skin	0.02
Used powdered pesticides on skin	0.14*
Wore pesticide-treated uniforms	0.04
Wore flea collars	0.19**
Saw living area sprayed/fogged with pesticides	0.14*
Received one or more shots in arm in theater	0.10
Received one or more shots in buttocks in theater	0.10
Used pyridostigmine bromide pills	−0.03
Contact with fresh CARC paint	0.13^†^

^†^*p* = 0.05, *p < 0.05, **p < 0.01. CARC, chemical agent resistant coating.

Because there was high degree of correlation and interrelationships among deployment-related exposures (data not shown), in post-hoc analyses, we used a backward stepwise linear regression model to examine the relationship between deployment-related exposures and VA-FI. The regression model was significant (*R*^2^ = 0.16, *F*_6,158_ = 5.07, *p* < 0.001). The final variables remaining in the model, that were significantly associated with VA-FI were Kansas GWI case status (standardized *β* = 0.26, *t* = 3.57, *p* < 0.001), wearing flea collar (standardized *β* = 0.15, *t* = 2.03, *p* < 0.05), and seeing living area sprayed or fogged with pesticides (standardized *β* = 0.15, *t* = 2.00, *p* < 0.05).

## 4. Discussion

To our knowledge, this is the first study to evaluate vulnerability to poor health outcomes in GW veterans using the frailty index as a proxy measure. Frailty is generally considered to be a state characterized by reduced physiological reserve and loss of resistance to stressors caused by accumulated age-related deficits (Clegg et al., 2013). Over the years, researchers have operationalized frailty in a couple of different ways. The frailty phenotype describes frailty as a biological syndrome with specific phenotypic presentations (e.g., unintentional weight loss, self-reported exhaustion, weakness, slow walking speed and low physical activity) (Fried et al., 2001). The frailty index operationalizes frailty as a state caused by the accumulation of health deficits during the life course (Mitnitski et al., 2001). Because the frailty index is calculated as a ratio of the number of deficits present to the number of total deficits considered, the more deficits one has, the more likely one is to be frail, the higher the frailty index. The VA-FI is an electronic index developed to use VHA administrative claims and electronic health records data to measure frailty specifically in Veterans (Orkaby et al., 2019).

The first major finding of this study is that GW veterans had lower VA-FIs (i.e., were healthier) than age- and sex-matched non-GW veterans. This finding was somewhat unexpected given previous reports of higher rates of chronic multisymptom illness, neurological, and other disorders in GW veterans compared to other veteran control groups (e.g., Ismail et al., 2008; Kang et al., 2009). However, there were more smokers among non-GW veterans compared to our GW veteran cohort. When we stratified the analysis by current smoking status, non-smoking non-GW veterans had marginally, but not significantly, higher VA-FIs compared to non-smoking non-GW veterans. Among smoking veterans, there was no significant difference between GW and non-GW veterans.

Because the two veteran groups were matched on age, it is likely a majority of the “non-GW” veterans are veterans of the U.S. wars in Afghanistan (Operation Enduring Freedom; OEF) and Iraq (Operation Iraqi Freedom; OIF; Operation New Dawn; OND). However, we could not verify this in VA records because all military personnel who served on active duty from August 2, 1990 to present are considered Gulf War veterans. This is because Congress never repealed the Authorization for Use of Military Force after the Gulf War ended in February 1991 (Affairs, 2021).

If, in fact, most of the “non-GW” veterans were OEF/OIF/OND veterans, one reason why this group of veterans had higher VA-FI than GW veterans may be due to traumatic brain injuries (TBI), the “signature injury” of the U.S. wars in Iraq and Afghanistan (Menon et al., 2010; Arriola and Rozelle, 2016). It is well documented that TBI can produce both acute and chronic consequences that lead to permanent disabilities that increase long-term mortality (Masel and DeWitt, 2010; Corrigan and Hammond, 2013) TBIs can also result in various secondary pathological conditions (Institute of Medicine, 2009). Because the changes initiated by TBI can persist for months to years after the initial injury (Bramlett and Dietrich, 2015), When we examined the individual VA-FI deficits, non-GW veterans had higher rates of anxiety, coronary artery disease, cerebrovascular disease, diabetes, hypertension, peripheral vascular disease, and weight loss than deployed GW veterans. It is noteworthy that a number of studies have reported increased risk for anxiety (Mallya et al., 2015), cardiovascular (Eric Nyam et al., 2019) and metabolic disorders (Kumar et al., 2018), including diabetes (Capatina et al., 2015) in the chronic phase of TBI recovery.

Another unexpected finding of this study is that rates of fatigue and chronic pain, two hallmark symptoms of GWI, were not higher in GW veterans compared to non-GW veterans at index date. However, not all of the GW veterans in the cohort had CMI/GWI at the time of the SFVHACS research studies. When we examined subgroups of GW veterans, we found differences in rates of chronic pain between controls and CDC CMI cases and between controls and Kansas GWI. Because the GW veterans were originally queried about their CMI/GWI symptoms 5–21 years prior to the index date, this suggests the veterans’ CMI/GWI symptoms of pain have not changed much over time. In contrast, rates of fatigue were not significantly different between controls and CMI cases or between controls and Kansas GWI cases. This suggests that, unlike pain, the veterans’ CMI/GWI symptoms of fatigue have likely changed or evolved over time.

Not all of the GW veterans in the retrospective cohort actually participated in research studies at the SFVAHCS. Some veterans were excluded because they did not meet the original studies’ inclusion criteria. Other veterans changed their minds and withdrew from the studies. Results indicate that veterans who were excluded from the original research studies had higher VA-FIs than veterans who were included and veterans who withdrew. This is not unexpected considering that the original SFVAHCS research studies’ exclusionary criteria included contraindications for MRI (e.g., pacemakers), severe physical impairment, psychiatric disorders with psychotic features, history of neurologic or systemic illnesses that affect central nervous system function, and/or moderate to severe traumatic brain injury. In fact, significantly more veterans who had been excluded from the original SFVAHCS research study were deceased at the index date compared to veterans who were included or who withdrew from SFVAHCS research studies.

There was a significant effect of CMI status on VA-FI: Veterans who met CDC CMI criteria at the time of the SFVAHCS studies had higher VA-FIs (i.e., were frailer) at the index date than controls (i.e., veterans who had insufficient symptoms to meet CMI criteria). Examination of individual VA-FI deficits revealed higher rates of anxiety, chronic pain, depression, lung disease, peripheral neuropathy, and dementia in CMI cases compared to controls. Considering that the mean age of the GW veteran cohort was 62 years at the index date, it is concerning that there was a higher rate of dementia (9.8% vs. 0.7%) among CMI cases compared to controls. We have previously reported a higher-than-expected rate of mild cognitive impairment (Chao, 2020), considered a prodromal phase of dementia (Petersen, 2011), in a subset of the GW veterans who are part of the current retrospective sample. Therefore, the current finding of a higher rates of dementia in CMI cases relative to controls lends further support to the idea that veterans with CMI are at increased risk for neurodegenerative diseases.

As with CDC CMI, there was a significant effect of Kansas GWI case status on VA-FI. As one might expect, GWI cases had higher VA-FIs than controls. However, veterans who were excluded from being considered as GWI cases because of medical and/or psychiatric conditions (i.e., GWI excludes) had even higher VA-FIs than GWI cases. When we examined individual VA-FI deficits, veterans who were excluded from being considered as GWI cases had higher rates of anemia, arthritis, coronary artery disease, depression, diabetes, hypertension, kidney disease, liver disease, peripheral neuropathy, and perivascular disease. This is not surprising because many of these conditions were likely what prevented these veterans from being considered as Kansas GWI cases at the time of the SFVAHCS studies. However, many of these conditions are also related to aging or may be accelerated by aging. Therefore, it is possible that not all of these medical conditions were pre-existing at the time of the SFVAHCS studies.

Several deployment-related experiences and exposures were significantly and positively correlated with VA-FI. These include hearing chemical alarms sound (a proxy measure for potential exposure to low levels of chemical nerve agent), using powdered pesticides, wearing flea collars, seeing ones’ living area sprayed or fogged with pesticides, and coming into contact with chemical agent resistant coating paint. Notably, most of these deployment-related experiences and exposures contain neurotoxins, particularly organophosphates. Previous studies have reported that many of the exposures associated with GW deployment are highly intercorrelated (Fricker et al., 2000; Cherry et al., 2001; Steele et al., 2012). In line with this, we also observed a high degree of correlation among deployment exposures in the GW veterans who completed the Kansas Gulf War and Health Questionnaire. Because this suggests a potential for confounding error when evaluating associations individually, in post-hoc analyses, we used a backward-step linear regression to examine the associations of deployment experiences and current health, as measured by VA-FI. After the least significant variables were removed, only Kansas GWI case status, wearing flea collars, and witnessing living are being sprayed or fogged with pesticides were significantly associated with VA-FI.

It is noteworthy that (Haley and Kurt, 1997) previously reported that wearing flea collars during the GW was associated with increased risk of developing Haley Syndrome 1 (impaired cognition). The Research Advisory Committee on Gulf War Veterans’ Illnesses (Research Advisory Committee on Gulf War Veterans Illnesses (Rac-Gwvi), 2008) has also cited evidence of an association between exposure to pesticides and multisymptom illness consistent with GWI. The current finding suggests that exposures to pesticides, particularly those present in flea collars, may be associated with frailty and current ill health independent of GWI.

This study had some limitations that warrant discussion. First, the cohort included only GW veterans who enrolled in research studies at the SFVAHCS and whose EHR were located in the VA’s CDW. This may limit the generalizability of these findings to the larger population of GW veterans. Presumably, the veterans whose EHR we were unable to locate in CDW were not utilizers of VA healthcare services. This may further limit the generalizability of these findings because it has been reported that VA users are more likely to have multiple medical conditions, endorse a disability, and score lower on measures of functioning than non-VA users (Meffert et al., 2019). Another limitation is the study’s reliance on self-reports, which is subject to recall bias, for health symptoms and deployment-related experiences and exposures. However, this limitation is common to other Gulf War research studies because there is no diagnostic test for GWI and self-report is currently the only method available for assessing GWI. Furthermore, because there was an absence of records or measurements quantifying chemical exposures during deployment, self-report is one of the only methods of investigating the effects of GW exposures on veteran health. It is possible that there were deployment-related exposures not assessed by the Kansas Military History and Health questionnaire that had an effect on the VA-FI. A final limitation is that the study was not adequately powered to examine potential sex differences in VA-FI and how biological sex may affect the relationship between exposures to deployment-related chemicals and current health status. Future, better powered studies will be necessary to address this question.

## 5. Conclusion

We evaluated GW veterans’ vulnerability to poor health outcomes using the frailty index as a proxy measure conclusion. Our results suggest that GW veterans, as a group, are not frailer (i.e., in worse health) than non-GW veterans who use VA healthcare. However, GW veterans who met criteria for severe CMI and who had Kansas GWI exclusionary conditions 5–21 years ago were significantly more frail than other GW veterans and non-GW veterans. We also found that GW veterans who met CDC CMI criteria had higher rates of dementia than control GW veterans. Thus, it may be prudent to counsel GW veterans with CMI to adopt lifestyle changes that have been associated with lowering dementia risk. The veteran subgroup with the highest VA-FI were GW veterans who had Kansas GWI exclusionary conditions. This suggests that research and treatment studies that employ the Kansas GWI case definition may be overlooking the subgroup of GW veterans who are most vulnerable to poor health outcomes.

## Data availability statement

The raw data supporting the conclusions of this article will be made available by the authors, without undue reservation.

## Ethics statement

The studies involving humans were approved by University of California, San Francisco Institutional Review Board. The studies were conducted in accordance with the local legislation and institutional requirements. The participants provided their written informed consent to participate in this study.

## Author contributions

LC designed the experiment, analyzed the data, drafted the manuscript, contributed to the article, and approved the submitted version.

## Funding

This study was supported by grant from the U.S. Department of Veterans Affairs (No. CX000798-05 and IK6CX002522).

## Conflict of interest

The author declares that the research was conducted in the absence of any commercial or financial relationships that could be construed as a potential conflict of interest.

## Publisher’s note

All claims expressed in this article are solely those of the authors and do not necessarily represent those of their affiliated organizations, or those of the publisher, the editors and the reviewers. Any product that may be evaluated in this article, or claim that may be made by its manufacturer, is not guaranteed or endorsed by the publisher.
